# Genome Sequence of *Pseudomonas koreensis* CRS05-R5, an Antagonistic Bacterium Isolated from Rice Paddy Field

**DOI:** 10.3389/fmicb.2016.01756

**Published:** 2016-11-08

**Authors:** Haiyan Lin, Shikai Hu, Ruifang Liu, Ping Chen, Changwei Ge, Bo Zhu, Longbiao Guo

**Affiliations:** ^1^State Key Lab for Rice Biology, China National Rice Research InstituteHangzhou, China; ^2^Agricultural Genomes Institute at Shenzhen, Chinese Academy of Agricultural SciencesShenzhen, China; ^3^Zhejiang Province Key Laboratory of Plant Secondary Metabolism and Regulation, College of Life Science, Zhejiang Sci-Tech UniversityHangzhou, China

**Keywords:** biocontrol, *Pseudomonas koreensis*, PacBio, secondary metabolism, unique gene

## Introduction

*Pseudomonas koreensis*, a new nominated Gram-negative bacterium was first reported and isolated from Korean agricultural soil (Kwon et al., 2003). CRS05-R5 (first reported as *Pseudomonas* sp.), which showed biocontrol ability against *Sitophilus oryzae* and *Acidovorax avenae* subsp. *avenae* (Liu et al., 2014), was first isolated from the rice rhizosphere in Heilongjiang province and reported in 2003 (Xie et al., 2003). Except for that, this species has been reported to produce the biosurfactant, which has biocontrol ability against *Phytophthora infestans* and *Pythium ultimum* (Hultberg et al., 2010a,b). These interesting features raise our attention on CRS05-R5. Recently, we sequenced the 16S rRNA sequence from CRS05-R5 and built the phylogenetic tree (Figure S1). Based on that, we confirmed that CRS05-R5 should be classified as *P. koreensis*. However, only one genome was sequenced (D26) and no detailed analysis was performed on this species. In this case, we did whole-genome sequencing on CRS05-R5, and tried to reveal the possible mechanism behind its antagonistic ability.

## Methods

### Genomic DNA isolation

Single colony of CRS05-R5 was inoculated into 5 ml NB (Nutrient Broth, BD, USA) at 30°C with 180 rpm vigorous shaking. 2.5 ml of culture broth was used to isolate the genomic DNA. DNA was extracted by Wizard Genomic DNA Purification Kit (Promega, Madison, WI, USA). The quality of purified genomic DNA was tested by using NanoDrop 2000 UV-Vis spectrophotometer (Thermo Scientific, MA, USA) and Qubit 2.0 fluorometer (Life Technologies, MA, USA), respectively.

### Whole genome sequencing and annotation

Whole genome sequencing of CRS05-R5 was carried out by using PacBio RS II platform. Six hundred Megabytes raw data was obtained with 100X coverage. After quality control, genome assembly was *de novo* assembled using HGAP assembly protocol, which is available with the SMRT Analysis packages and accessed through the SMRT Analysis Portal version 2.1. Genome annotation was later done by using RAST annotation system (Overbeek et al., 2014). The completeness of the assembled genome was tested by CheckM with default parameters (Parks et al., 2015). In addition, GO and COG programs were used to do further functional analysis of all annotated ORFs (Ashburner et al., 2000; Tatusov et al., 2000). Specifically, since antagonistic bacteria, especially fluorescent pseudomonads, usually compete with other microorganisms by using secondary metabolism (Haas and Défago, 2005), genes, which are predicted to be involved in secondary metabolism were compared with DoBISCUIT database (Ichikawa et al., 2013). The circular genome map of CRS05-R5 including all predicted ORFs with COG functional assignments, rRNA, tRNA, G+C content, and GC skew information were generated using Circos (Krzywinski et al., 2009), as shown in Figure S2.

### Genome comparison

Genome comparison among CRS05-R5 and other fully sequenced *Pseudomonas* genomes were carried out by using ANI (average nucleotide identity) and AF (alignment fraction), which were calculated by ANIcalculator (Varghese et al., 2015), and Circos (Krzywinski et al., 2009). In order to find out the unique genes in CRS05-R5, all the fully sequenced *Pseudomonas* genomes were downloaded from NCBI (58 genomes). The protein sequences from CRS05-R5 were compared with the protein sequences from these 58 genomes with 40% identity as cutoff. The protein sequences which cannot find any homologs in these 58 genomes were classified as unique genes.

### Direct link to deposited data and information to users

This strain has been deposited in CGMCC with deposit number 1.15630. This genome sequencing project has been deposited at DDBJ/EMBL/GenBank under the Accession Number CP015852. The BioProject designation for this project is PRJNA322127.

## Interpretation of data set

### General genome sequence property

The total size of the genome is 5,991,224 bp and has a G+C content of 60.6%. The completeness and contamination of this genome is 99.86% and 0.05% after running the CheckM. These results indicate the high quality of assembled genome. A total of 5352 CDSs were predicted. Of these, 3832 could be assigned to a COG number. The most abundant COG category was “General function prediction only” (581 proteins) followed by “Signal transduction mechanisms” (547 proteins), “Amino acid transport and metabolism” (539 proteins), “Transcription” (448 proteins), and “Function unknown” (302 proteins). In addition, 82 RNAs including rRNA and tRNA were identified. All the genomic information was shown in Table 1.

**Table 1:** **Genome statistics**.

**Features**	**Value**
Genome size(bp)	5,991,224
Contig numbers	1
G+C %	60.6
Protein-coding genes	5352
Protein with known function	4363
tRNA number	76
rRNA number	6
ncRNA number	75
Genes with signal peptides	573
Genes with transmembrane helices	1187

### Secondary metabolism in CRS05-R5

Biosurfactants are amphiphilic compounds produced by microorganisms (Mulligan, 2005). This compound, which is produced by *Pseudomonas* spp., has been widely used in biocontrol (Debode et al., 2007). In CRS05-R5 genome, more than 800 genes are predicted to be involved in secondary metabolism by comparing with DoBISCUIT database (Ichikawa et al., 2013). Interestingly, we found one gene cluster, which is annotated as cyclic lipopeptides (CLPs), exists in CRS05-R5 (*arfABC*). CLP is a kind of biosurfactant, and has been proved to be important in antagonistic *Pseudomonas* sp. (Raaijmakers et al., 2006). This information indicates that CRS05-R5 can be used as biocontrol agent.

### Genome comparison

**Figure 1:**
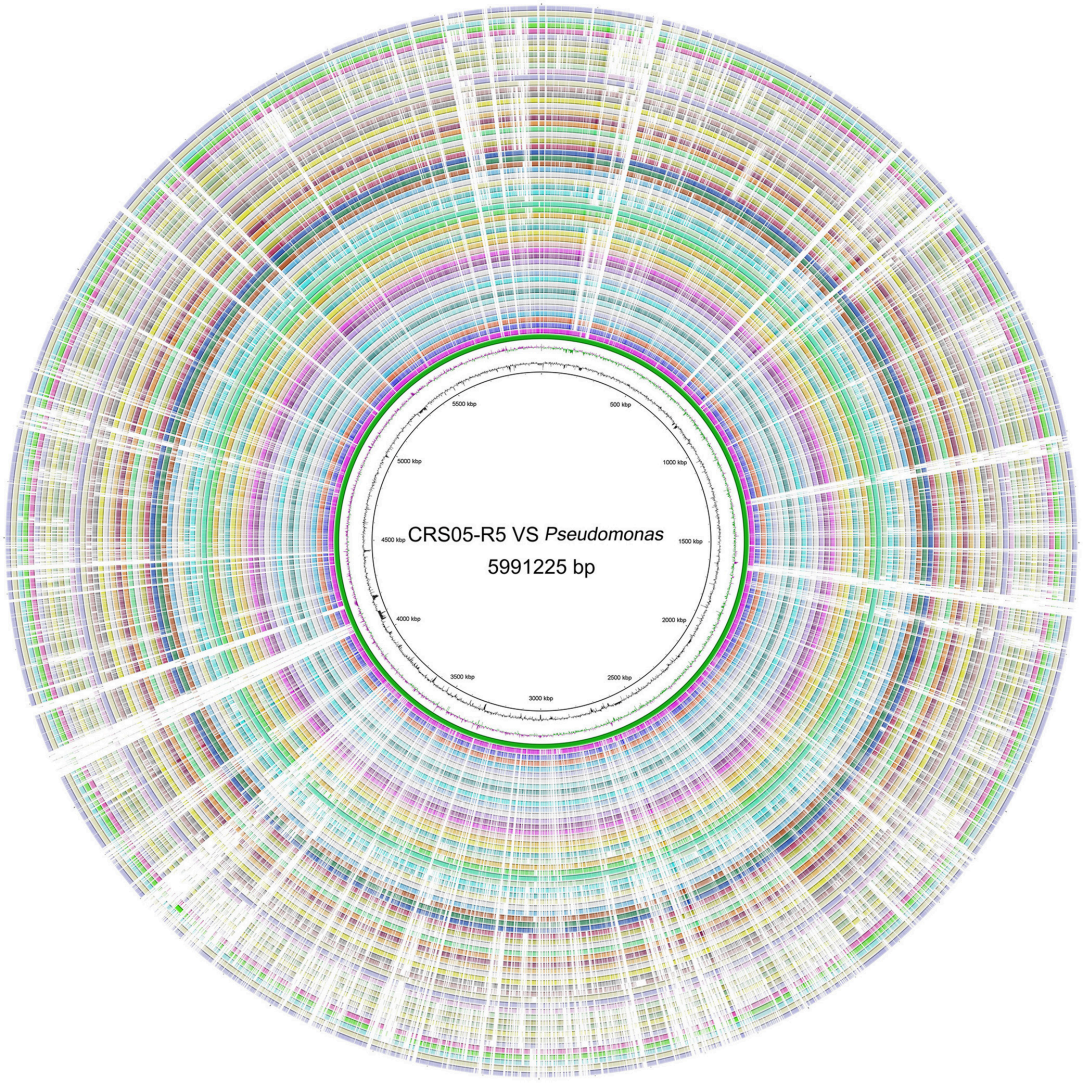
**Circular map of the chromosome of *P. koreensis* CRS05-R5 and other fully sequenced *Pseudomonas* genomes**. The tracks from the inside to outside: GC Content, GC Skew, *P. koreensis* CRS05-R5, *P. koreensis* D26, *P. aeruginosa* B136-33, *P. aeruginosa* c7447 m, *P. aeruginosa* DK2, *P. aeruginosa* LES431, *P. aeruginosa* LESB58, *P. aeruginosa* M18, *P. aeruginosa* MTB-1, *P. aeruginosa* NCGM2.S1, *P. aeruginosa* PA1, *P. aeruginosa* PA1R, *P. aeruginosa* PA7, *P. aeruginosa* PAO1-VE13, *P. aeruginosa* PAO1-VE2, *P. aeruginosa* PAO1, *P. aeruginosa* PAO581, *P. aeruginosa* RP73, *P. aeruginosa* SCV20265, *P. aeruginosa* UCBPP-PA14, *P. denitrificans* ATCC 13867, *P. entomophila* L48, *P. fluorescens* A506, *P. fluorescens* Pf0-1, *P. fluorescens* SBW25, *P. fulva* 12-X, *P. mendocina* NK-01, *P. mendocina* ymp, *P. monteilii* SB3078, *P. monteilii* SB3101, *P. poae* RE1-1-14, *P. protegens* CHA0, *P. protegens* Pf-5, *P. putida* DOT-T1E, *P. putida* F1, *P. putida* GB-1, *P. putida* H8234, *P. putida* HB3267, *P. putida* KT2440, *P. putida* NBRC 14164, *P. putida* ND6, *P. putida* S16, *P. putida* W619, *P. resinovorans* NBRC 106553, *Pseudomonas* sp. TKP, *Pseudomonas* sp. UW4, *Pseudomonas* sp. VLB120, *P. stutzeri* A1501, *P. stutzeri* ATCC 17588, *P. stutzeri* CCUG 29243, *P. stutzeri* DSM 10701, *P. stutzeri* DSM 4166, *P. stutzeri* RCH2, *P. syringae* pv. *phaseolicola* 1448A, *P. syringae* pv. *syringae* B728a, *P. syringae* pv. tomato DC3000, *P. brassicacearum* subsp. *Brassicacearum* NFM421, *P. fluorescens* F113.

Strikingly, we found even the ANI value between CRS05-R5 and *P. koreensis* D26 is only 92.5% (Figure S3). Also, the highest AF value is only 74.2% (Table S1). Indeed, 631 CDSs were predicted to be unique genes existing in CRS05-R5 genome. These results indicate the huge genome diversity among *Pseudomonas* strains, which is consistent with previous findings (Loper et al., 2012). Circos result found many unique genes in CRS05-R5 (Figure 1). To better understand the features of these genes, *Pseudomonas* database was used to deeply annotate these genes (Winsor et al., 2011). Except for a lot of hypothetical proteins, we found one gene cluster encoding fimbrial associated proteins only existing in CRS05-R5 (A8L59_09240–A8L59_09310). These genes have been reported to be critical for the initial stage of biofilm development (Wei and Ma, 2013). Except for this gene cluster, we also found three rhs genes exist as the unique genes in CRS05-R5 (A8L59_00750, A8L59_09890, and A8L59_11585). These genes have been found to be linked to the second type VI secretion cluster in *P. aeruginosa* (Jones et al., 2014). Also, these genes have been reported to mediate intercellular competition (Koskiniemi et al., 2013). More strikingly, one rhs gene is located in a unique gene cluster (A8L59_11555–A8L59_11600). Although, most of the genes are hypothetical proteins, we may infer that this cluster maybe related with secretions. Indeed, by searching with TMHMM Server v. 2.0, which is a transmembrane helices prediction database (http://www.cbs.dtu.dk/services/TMHMM/), we found that 3 genes (A8L59_11560, A8L59_11565, and A8L59_11600) have predicted transmembrane helices. Since biofilm and intercellular competition are very important for bacterial survival and adaptation, we infer that these genes may confer some fitness advantages for CRS05-R5 on the root of *Oryza sativa*. Also, we found one unique gene cluster (A8L59_18500–A8L59_18575), which encodes wbpL and other glycosyl transferase. These genes have been confirmed to be important in lipopolysaccharide formation (Rocchetta et al., 1998). These compounds have been proved to be important in plant roots colonization (Duijff et al., 1997) as well as induction of systemic resistance against some plant pathogens (Leeman et al., 1995). All these results strongly indicate the biocontrol potential of CRS05-R5 strain.

## Author contributions

HL performed the experiments and write the manuscript. SH and RL helped in data analysis. PC, CG, and BZ coordinated the work and drafted the manuscript. LG conceived the work.

## Funding

This work was supported by grants from the Natural Science Fund of China (grant No. 31521064), and the Chinese 973 Program (2013CBA01405).

### Conflict of interest statement

The authors declare that the research was conducted in the absence of any commercial or financial relationships that could be construed as a potential conflict of interest.

## Supplementary material

Table S1 **The fraction of orthologous genes (Alignment Fraction, AF) value between CRS05-R5 and other Pseudomonas genomes**.

Figure S1 **Phylogenetic relationships based on 16S rRNA gene sequences were determined by the neighbor-joining method with the program package MEGA 6.0 (Tamura et al., 2013)**. Bootstrap confidence values were obtained using 1000 resamplings. The tree shows the positions of strains CRS05-R5 and other selected *Pseudomonas* strains. Numbers at nodes indicate percentages of occurrence in 1000 bootstrapped trees; only values >50% are shown. Bar, 0.005 substitutions per site.

Figure S2 **Graphical map of the chromosome genome of *P. koreensis* CRS05-R5**. From the outside to the center: genes on forward stand and Genes on reverse strand (color by COG categories), RNA genes (tRNAs green, rRNAs red, ncRNAs black), GC content, GC skew.

Figure S3 **Heatmap of Average Nucleotide Identity (ANI) between CRS05-R5 and all the other sequenced *Pseudomonas* genomes**.

## References

[B1] Ashburner M. Ball C. A. Blake J. A. Botstein D. Butler H. Cherry J. M.. (2000). Gene ontology: tool for the unification of biology. Nat. Genet. 25, 25–29. 10.1038/75556 10802651 PMC3037419

[B2] Debode J. De Maeyer K. Perneel M. Pannecoucque J. De Backer G. Höfte M. (2007). Biosurfactants are involved in the biological control of *Verticillium microsclerotia* by *Pseudomonas* spp. J. Appl. Microbiol. 103, 1184–1196. 10.1111/j.1365-2672.2007.03348.x 17897223

[B3] Duijff B. J. Gianinazzi-Pearson V. Lemanceau P. (1997). Involvement of the outer membrane lipopolysaccharides in the endophytic colonization of tomato roots by biocontrol *Pseudomonas fluorescens* strain WCS417r. New Phytol. 135, 325–334. 10.1046/j.1469-8137.1997.00646.x

[B4] Haas D. Défago G. (2005). Biological control of soil-borne pathogens by fluorescent pseudomonads. Nat. Rev. Microbiol. 3, 307–319. 10.1038/nrmicro1129 15759041

[B5] Hultberg M. Alsberg T. Khalil S. Alsanius B. (2010a). Suppression of disease in tomato infected by *Pythium ultimum* with a biosurfactant produced by *Pseudomonas koreensis*. Biocontrol 55, 435–444. 10.1007/s10526-009-9261-6

[B6] Hultberg M. Bengtsson T. Liljeroth E. (2010b). Late blight on potato is suppressed by the biosurfactant-producing strain *Pseudomonas koreensis* 2.74 and its biosurfactant. Biocontrol 55, 543–550. 10.1007/s10526-010-9289-7

[B7] Ichikawa N. Sasagawa M. Yamamoto M. Komaki H. Yoshida Y. Yamazaki S.. (2013). DoBISCUIT: a database of secondary metabolite biosynthetic gene clusters. Nucleic Acids Res. 41, D408–D414. 10.1093/nar/gks1177 23185043 PMC3531092

[B8] Jones C. Hachani A. Manoli E. Filloux A. (2014). An rhs gene linked to the second type vi secretion cluster is a feature of the *Pseudomonas aeruginosa* strain PA14. J. Bacteriol. 196, 800–810. 10.1128/JB.00863-13 24317402 PMC3911176

[B9] Koskiniemi S. Lamoureux J. G. Nikolakakis K. C. t'Kint de Roodenbeke C. Kaplan M. D. Low D. A.. (2013). Rhs proteins from diverse bacteria mediate intercellular competition. Proc. Natl. Acad. Sci. U.S.A. 110, 7032–7037. 10.1073/pnas.1300627110 23572593 PMC3637788

[B10] Krzywinski M. Schein J. Birol I. Connors J. Gascoyne R. Horsman D.. (2009). Circos: an information aesthetic for comparative genomics. Genome Res. 19, 1639–1645. 10.1101/gr.092759.109 19541911 PMC2752132

[B11] Kwon S. W. Kim J. S. Park I. C. Yoon S. H. Park D. H. Lim C. K.. (2003). *Pseudomonas koreensis* sp. nov., *Pseudomonas umsongensis* sp. nov. and *Pseudomonas jinjuensis* sp. nov., novel species from farm soils in Korea. Int. J. Syst. Evol. Microbiol. 53, 21–27. 10.1099/ijs.0.02326-0 12656147

[B12] Leeman M. Van Pelt J. Den Ouden F. Heinsbroek M. Bakker P. Schippers B. (1995). Induction of systemic resistance against *Fusarium* wilt of radish by lipopolysaccharides of *Pseudomonas fluorescens*. Phytopathology 85, 1021–1027. 10.1094/Phyto-85-1021

[B13] Liu H. Yang C. L. Ge M. Y. Ibrahim M. Li B. Zhao W. J.. (2014). Regulatory role of tetR gene in a novel gene cluster of *Acidovorax avenae* subsp. *avenae* RS-1 under oxidative stress. Front. Microbiol. 5:547. 10.3389/fmicb.2014.00547 25374564 PMC4204640

[B14] Loper J. E. Hassan K. A. Mavrodi D. V. Davis E. W. II Lim C. K. Shaffer B. T.. (2012). Comparative genomics of plant-associated *Pseudomonas* spp.: insights into diversity and inheritance of traits involved in multitrophic interactions. PLoS Genet. 8:e1002784. 10.1371/journal.pgen.1002784 22792073 PMC3390384

[B15] Mulligan C. N. (2005). Environmental applications for biosurfactants. Environ. Pollut. 133, 183–198. 10.1016/j.envpol.2004.06.009 15519450

[B16] Overbeek R. Olson R. Pusch G. D. Olsen G. J. Davis J. J. Disz T.. (2014). The SEED and the Rapid Annotation of microbial genomes using Subsystems Technology (RAST). Nucleic Acids Res. 42, D206–D214. 10.1093/nar/gkt1226 24293654 PMC3965101

[B17] Parks D. H. Imelfort M. Skennerton C. T. Hugenholtz P. Tyson G. W. (2015). CheckM: assessing the quality of microbial genomes recovered from isolates, single cells, and metagenomes. Genome Res. 25, 1043–1055. 10.1101/gr.186072.114 25977477 PMC4484387

[B18] Raaijmakers J. M. de Bruijn I. de Kock M. J. D. (2006). Cyclic lipopeptide production by plant-associated *Pseudomonas* spp.: diversity, activity, biosynthesis, and regulation. Mol. Plant. Microbe Interact. 19, 699–710. 10.1094/MPMI-19-0699 16838783

[B19] Rocchetta H. L. Burrows L. L. Pacan J. C. Lam J. S. (1998). Three rhamnosyltransferases responsible for assembly of the A-band D-rhamnan polysaccharide in *Pseudomonas aeruginosa*: a fourth transferase, WbpL, is required for the initiation of both A-band and B-band lipopolysaccharide synthesis. Mol. Microbiol. 28, 1103–1119. 10.1046/j.1365-2958.1998.00871.x 9680202

[B20] Tamura K. Stecher G. Peterson D. Filipski A. Kumar S. (2013). MEGA6: molecular evolutionary genetics analysis version 6.0. Mol. Biol. Evol. 30, 2725–2729. 10.1093/molbev/mst197 24132122 PMC3840312

[B21] Tatusov R. L. Galperin M. Y. Natale D. A. Koonin E. V. (2000). The COG database: a tool for genome-scale analysis of protein functions and evolution. Nucleic Acids Res. 28, 33–36. 10.1093/nar/28.1.33 10592175 PMC102395

[B22] Varghese N. J. Mukherjee S. Ivanova N. Konstantinidis K. T. Mavrommatis K. Kyrpides N. C.. (2015). Microbial species delineation using whole genome sequences. Nucleic Acids Res. 43, 6761–6771. 10.1093/nar/gkv657 26150420 PMC4538840

[B23] Wei Q. Ma L. Z. (2013). Biofilm matrix and its regulation in Pseudomonas aeruginosa. Int. J Mol. Sci. 14, 20983–21005. 10.3390/ijms141020983 24145749 PMC3821654

[B24] Winsor G. L. Lam D. K. Fleming L. Lo R. Whiteside M. D. Nancy Y. Y.. (2011). Pseudomonas genome database: improved comparative analysis and population genomics capability for *Pseudomonas* genomes. Nucleic Acids Res. 39, D596–D600. 10.1093/nar/gkq869 20929876 PMC3013766

[B25] Xie G. L. Soad A. Swings J. Mew T. (2003). Diversity of Gram negative bacteria antagonistic against major pathogens of rice from rice seed in the tropic environment. J. Zhejiang Univ. Sci. A 4, 463–468. 10.1631/jzus.2003.0463 12861624

