# Supplementary figures and images for: Genome Sequence of Pseudomonas koreensis CRS05-R5, an Antagonistic Bacterium Isolated from Rice Paddy Field
Source: Front Microbiol. 2016 Nov 8;7:1756. doi: 10.3389/fmicb.2016.01756 (PMC5099245; doi:10.3389/fmicb.2016.01756)

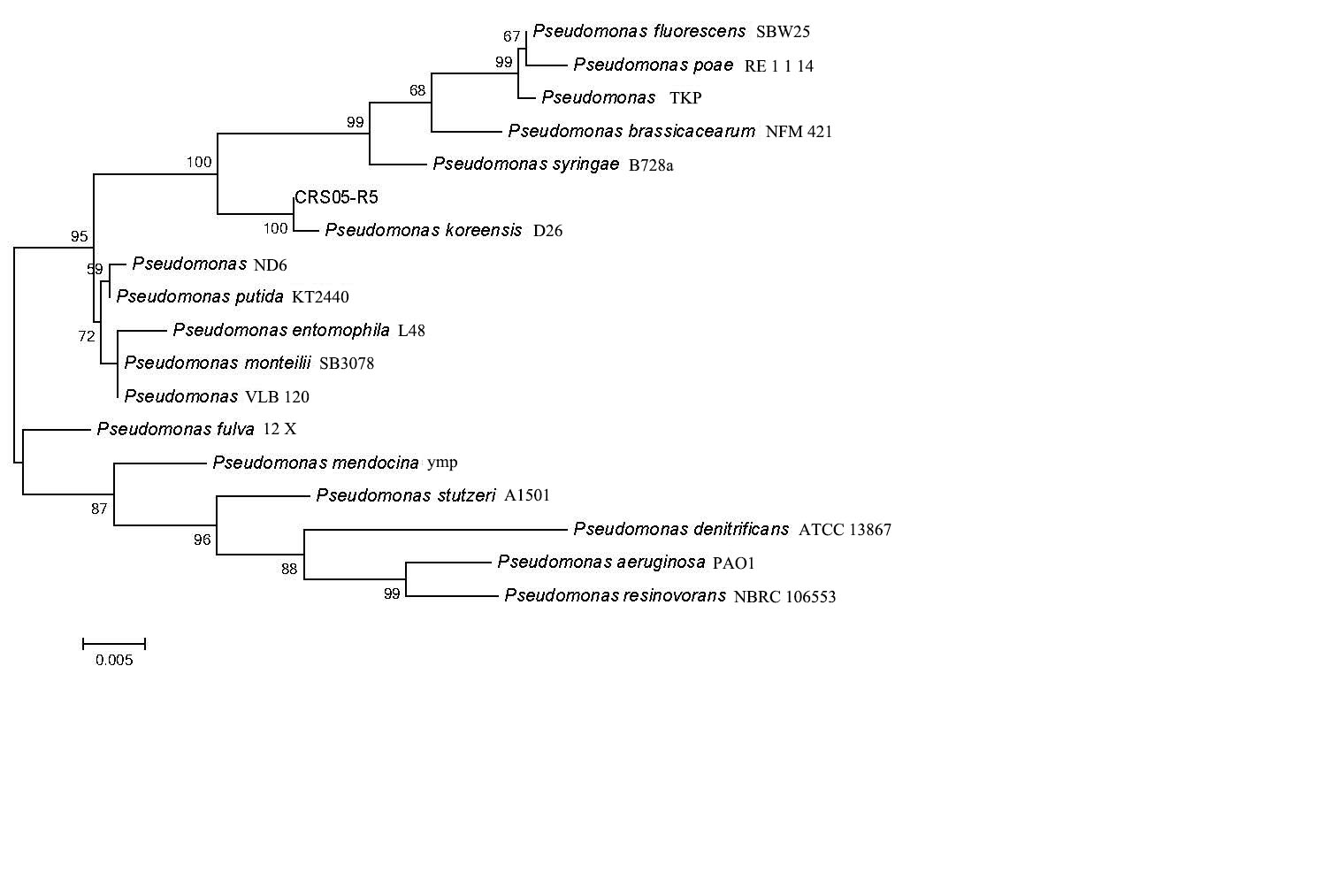

Supplement: Figure S1 — Phylogenetic relationships based on 16S rRNA gene sequences were determined by the neighbor-joining method with the program package MEGA 6.0 (Tamura et al., 2013). Bootstrap confidence values were obtained using 1000 resamplings. The tree shows the positions of strains CRS05-R5 and other selected Pseudomonas strains. Numbers at nodes indicate percentages of occurrence in 1000 bootstrapped trees; only values >50% are shown. Bar, 0.005 substitutions per site. [file Image1.JPEG]

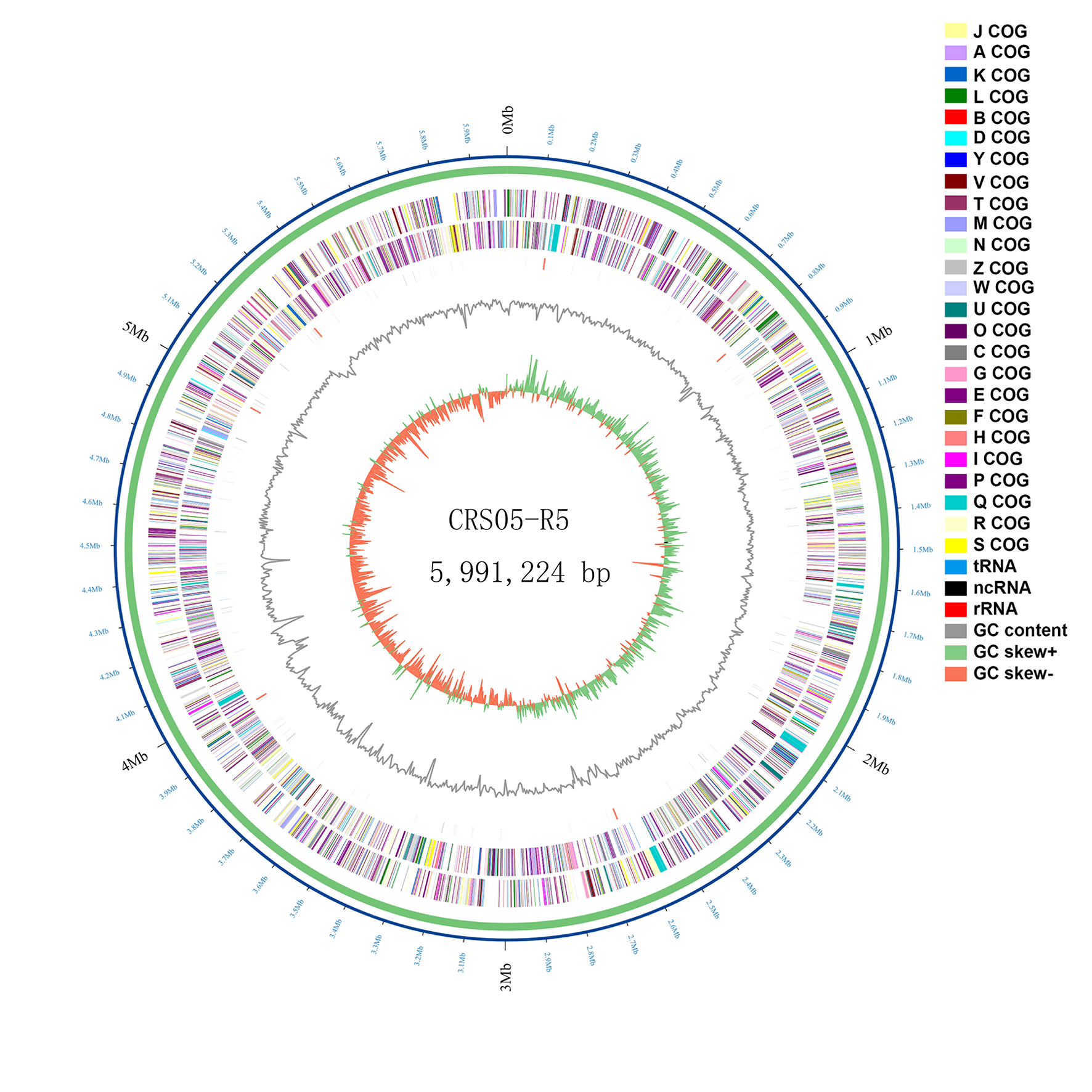

Supplement: Figure S2 — Graphical map of the chromosome genome of P. koreensis CRS05-R5. From the outside to the center: genes on forward stand and Genes on reverse strand (color by COG categories), RNA genes (tRNAs green, rRNAs red, ncRNAs black), GC content, GC skew. [file Image2.TIF]

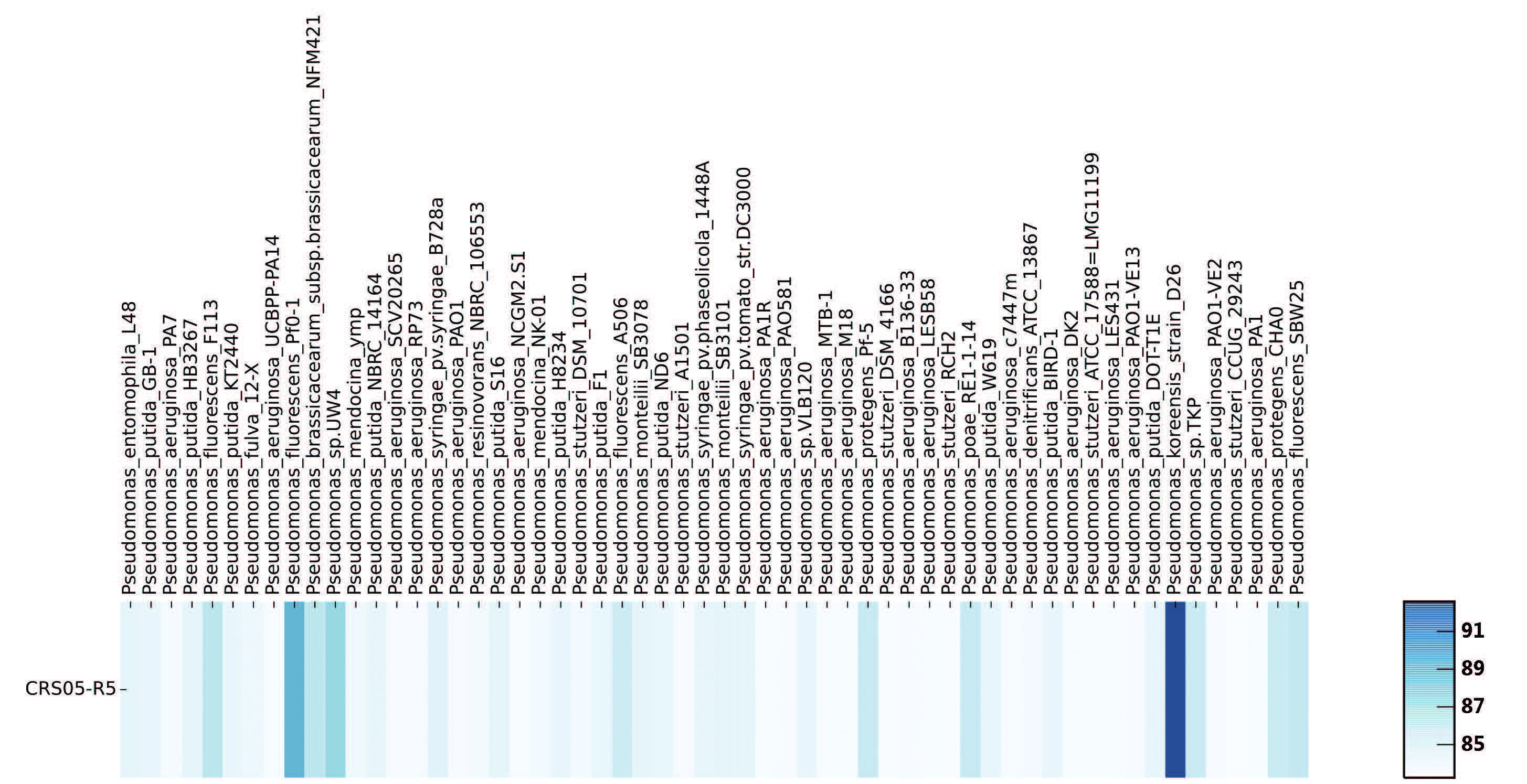

Supplement: Figure S3 — Heatmap of Average Nucleotide Identity (ANI) between CRS05-R5 and all the other sequenced Pseudomonas genomes. [file Image3.JPEG]
